# PNO1 promotes the progression of osteosarcoma via TGF-β and YAP/TAZ pathway

**DOI:** 10.1038/s41598-023-49295-8

**Published:** 2023-12-09

**Authors:** Long Fang, Baolong Wang, Zengkun Yang, Tingbao Zhao, Wei Hao

**Affiliations:** 1 Department of Bone and Soft Tissue Tumors, Shandong Provincial Third Hospital, Shandong University, Jinan, 250000 China; 2 Department of Orthopedics and Traumatology, Shandong Provincial Third Hospital, Shandong University, Jinan, 250000 China

**Keywords:** Biochemistry, Cancer

## Abstract

This study aimed to explore the potential role and mechanisms of the partner of NOB1 homolog (PNO1) in osteosarcoma. The expression of PNO1 in tumor and adjacent tissue samples was examined using western blotting. Lentiviral transfection was used to establish sh-Ctrl and sh-PNO1 osteosarcoma cell lines. MTT assay, Celigo cell cytometer count, and cell colony formation assay were used to investigate the proliferation of osteosarcoma cells in vitro, whereas xenotransplantation assay was performed for in vivo experiments. Wound-healing and Transwell assays were chosen to verify the migration and invasion of osteosarcoma cells. Flow cytometry assay and caspase-3/7 activity analysis were adopted for the analysis of cell apoptosis and cell cycle. Finally, transcriptome sequencing and bioinformatics analysis were adopted to explore the acting mechanisms. The expression of PNO1 was higher in osteosarcoma tissues than that in adjacent tissues. Down-regulation of PNO1 inhibited the proliferation, migration, and invasion, and induced cell apoptosis and cell cycle arrest of osteosarcoma cells. Furthermore, according to transcriptome sequencing and Kyoto Encyclopedia of Genes and Genomes pathway analysis, we found that PNO1 might affect the progression of osteosarcoma via TGF-β and YAP/TAZ signaling pathways. PNO1 could be a potential target for osteosarcoma treatment.

## Introduction

Osteosarcoma is recognized as the most common primary malignant bone tumor in children and adolescents, which usually occurs in the metaphysis of long bones^1,2^. In the last 40 years, systemic chemotherapy combined with surgery has significantly improved the outcomes in patients with osteosarcoma, and about 70% of patients without metastasis have long-term survival. However, the survival rate in patients with metastasis is quite low, with the five-year survival of only approximately 20%^3^. Recently, improved molecular biology technology provides new insights into the pathology and therapy of osteosarcoma. Several target therapies such as monoclonal antibodies, antibody–drug conjugates, and immune-checkpoint inhibitors become hotspots for osteosarcoma therapy^4^. Despite studies on existing therapeutic targets, searching for new potential therapeutic targets for osteosarcoma bears significance and needing urgent attention.

Human RNA binding gene partner of NOB1 (PNO1) was firstly isolated from human kidney and encodes 35-kDa protein, named PNO1^5^. PNO1 as a ribosome biogenesis factor that contributes to 40S subunit assembly by binding with its partner NOB1^6^. Recently, emerging evidences suggest the crucial regulation of PNO1 in human cancer. PNO1 could alter tumorigenesis through mediating multiple oncogenic signaling pathways. The over-expression of PNO1 was found in lung adenocarcinoma through the analysis based on bioinformatics databases and clinical investigation. Moreover, PNO1 exerted tumor promoting action in lung adenocarcinoma through activating Notch pathway, which was involved in cancer metastasis through modulating epithelial mesenchymal transition (EMT)^7^. PNO1 was confirmed as a negative regulator of p53 pathway, and its excessive expression resulted in enhanced cell viability through suppressing cell apoptosis^8^. The deletion of PNO1 significantly reduced the viability and motility of esophageal cancer cells, and PNO1 might played promoting roles in this cancer progression through mediating AKT1, Twist, Myc, mTOR, MMP2, NF-κB p65 and CTNNB1 expression^9^. PNO1 also had great regulatory function in tumor metabolism. The study performed by Hu et al. found that PNO1 could suppress ferroptosis of hepatocellular carcinoma through enhancing glutathione (GSH) metabolism^10^. These studies showed the potential of PNO1 to serve as a candidate for cancer target therapy. However, the study regarding the function and mechanisms of PNO1 in osteosarcoma is limited.

In this study, we aimed to examine the PNO1 expression in osteosarcoma samples, explore the effect of PNO1 in vivo and in vitro. In addition, according to the transcriptome sequencing and bioinformatics analysis, we elucidated the oncogenic mechanism of PNO1 in osteosarcoma cells.

## Materials and methods

### Patients and tissue samples

Six pairs of surgically resected cancer and adjacent normal tissue samples were collected from six osteosarcoma patients in the Third Hospital of Shandong province. The cancerous specimens were collected from the tumor lesions, while the adjacent tissues were taken from outside the surgical margin (3–5 cm away from the cancerous lesions). All the collected tissues were verified by pathological evaluation.

All the patients provided their informed consent prior to their inclusion in the study. The sample collection was carried out following the ethical guidelines of the Helsinki Declaration.

### Cell culture

The human osteosarcoma cell lines MNNG/HOS and U2OS were purchased from Wuhan Procell Life and Technology (Wuhan, China). The cells were cultured in DMEM (HyClone; Thermo Fisher Scientific) supplemented with 10% FBS (Gibco; Thermo Fisher Scientific) at 37 °C with 5% CO_2_.

#### PNO1 knockdown

sh-PNO1 (TGAACAATTTCAGTCATTT) and sh-Ctrl (TTCTCCGAACGTGTCACGT) were obtained from Shanghai Genechem (Shanghai, China). GV115 was employed as the lentiviral vector. MNNG/HOS and U2OS cells were seeded into 6-well plates and incubated at 37 °C with the presence of 5% CO_2_. 24 h later, MNNG/HOS and U2OS cells were transfected with PNO1-shRNA and control shRNA following the manufacturer’s protocol. The transfected cells were incubated for additional 72 h, and then harvested for further analysis. The efficiency of PNO1 knockdown was characterized via qRT-PCR and western blotting.

#### MTT cell proliferation assay

MTT (Cat. No. JT343; Genview, USA) assay was used to verify the effect of PNO1 knockdown on cell proliferation. The cells at the logarithmic growth phase were first trypsinized and then seeded into a 96-well plate with 1.5 × 10^3^ cells per well for MNNG/HOS cells and 2 × 10^3^ cells per well for U2OS cells. After incubation for 24, 48, 72, 96, and 120 h, 20 μL of 5 mg/mL MTT was added, and incubated for 4 h, and then the culture solution was completely aspirated and 100 μL of dimethyl sulfoxide (Cat. No. 130701; Shanghai Trial Chemical Reagent Co., Ltd., Shanghai, China) was added to dissolve formazan. The OD value at 490 nm was measured to evaluate the density of the cultured cells.

#### Celigo cell count

sh-PNO1 and sh-Ctrl cells were seeded into 96-well plates, with 1.5 × 10^3^ cells per well for MNNG/HOS cells and 1 × 10^3^ cells per well for U2OS cells. After 24 h, a Celigo Image Cytometer (Nexcelom, USA) was used to measure the number of green fluorescent cells in each plate once a day for 5 days.

#### Cell colony formation assay

sh-PNO1 and sh-Ctrl cells were planted in 6-well plates at a density of 1000 cells per well and cultured in complete medium. After 10 days, the cells were fixed with 4% paraformaldehyde (Sinopharm Chemical Reagent Co., Ltd., Beijing, China) at room temperature for 30 min and stained with 500 μL of Giemsa (Cat. No. AR-0752; Shanghai Ding Guo, China) at room temperature for 20 min. Then, the newly formed colonies were imaged and counted.

#### Flow cytometry cell apoptosis assay

Apoptosis was detected using an Annexin V-PE/7-AAD apoptosis detection kit (Cat. No. MA0429, Meilunbio, China) following the manufacturer's protocols. sh-PNO1 and sh-Ctrl cells were trypsinized, resuspended in complete medium, and centrifuged at 2000 rpm for 3 min. Then, the supernatant was discarded. The cell pellet was washed with pre-cooled phosphate-buffered saline (PBS) at 4 °C and then centrifuged at 2000 rpm for 3 min. The cell pellet was resuspended in 300 μL of 1 × binding buffer, stained with 5 μL of Annexin V-PE, protected from light for 10–15 min at room temperature, then stained with 5 μL of PI for 5 min, and mixed with 200 μL of 1 × binding buffer before measurement.

#### Caspase-3/7 activity analysis

sh-PNO1 and sh-Ctrl cells were seeded in 96-well plates and incubated at 37 °C in a 5% CO_2_ incubator for 72 h. Caspase-Glo 3/7 Assay (Cat. No. G8091; Promega, USA) reaction solution was prepared following the manufacturer’s protocols. The cells were collected and seeded in new 96-well plates. Further, 100 μL of Caspase-Glo 3/7 Assay reaction solution was added per well. The plates were then incubated at room temperature for 1–2 h, and then the fluorescence intensity was measured and analyzed.

#### Flow cytometry cell cycle assay

The cell cycle was detected using a cell cycle and apoptosis analysis kit (Cat. No. MA0334; Meilunbio, China) following the manufacturer's protocols. sh-PNO1 and sh-Ctrl cells were trypsinized, resuspended in complete medium, and centrifuged at 1300 rpm for 5 min. Then, the supernatant was discarded. The cell pellet was washed with pre-cooled D-Hanks solution (Shanghai Genechem Co., Ltd., Shanghai, China) 4 °C and then centrifuged at 1300 rpm for 5 min. The cell pellet was fixed with pre-cooled 75% ethyl alcohol at 4 °C for 1 h. After the cells were centrifuged and washed following the aforementioned steps, 1 mL of cell staining solution was added. A NovoCyte flow cytometer (Agilent, USA) was employed to detect cell cycle.

#### Wound-healing assay

The cells were seeded into 6-well plates (1 × 10^6^ cells/well). Then, a pipette tip was used to create a scratch on the cell surface at 90% confluence. The cells were rinsed with PBS to remove all cellular debris. After 0, 24, and 48 h of scratch creation, the cells were observed and imaged under a microscope (Olympus, Japan).

#### Transwell assay

We used 24-well Transwell insert chambers (Cat. No. 3422; Corning, USA) with the filtration membrane pore size of 8 µm to verify the cell migration and invasion. For invasion assays, the upper chambers were coated with Matrigel and incubated for 2 h before the cells were seeded. sh-PNO1 and sh-Ctrl cells with 100 μL of serum-free medium were seeded into the upper chambers, while the medium supplemented with 600 μL of 10% FBS was added to the lower chambers. After 24 h of incubation, the non-migrated or non-invaded cells were removed using a cotton swab, and the migrated or invaded cells were fixed with 4% paraformaldehyde and stained with crystal violet. Then, the cells were observed and imaged under the microscope.

#### In vivo xenotransplantation assay

A total of 20 4-week-old BALB/c female nude mice were equally divided into sh-Ctrl and sh-PNO1 groups. sh-PNO1 and sh-Ctrl MNNG/HOS cells were subcutaneously injected into the right arm pit of each mouse corresponding to the group. The length and width of tumors were measured every week until week 4. All mice were killed via injecting an overdose of 2% pentobarbital sodium followed by cervical vertebra dislocation. Then, the tumors were harvested and measured for volume and weight. This animal experiment was approved by the Ethics of Animal Experiments of the Third Hospital of Shandong province (approval no. KYLL-2022121), in compliance with the International Association of Veterinary Editors on the Author's Guide on Animal Ethics and Welfare. All the methods and treatments were in accordance with ARRIVE guidelines (https://arriveguidelines.org). All the animal operations were under anesthesia, and we make every effort to minimize their pain and death.

### Transcriptome sequencing and bioinformatics analysis

sh-PNO1 and sh-Ctrl MNNG/HOS cells (three samples in each group) were employed for transcriptome sequencing. Total RNA was extracted using the TRIzol reagent and examined with the Agilent 2100 Bioanalyzer system and Agilent 2100 RNA Nano 6000 Assay Kit (Agilent) following the manufacturer's protocols. Then, total transcriptome was sequenced based on the Illumina platform following a PE150 sequencing strategy. Differentially expressed genes (DEGs) were defined as | log_2_ Fold change |> 2 and q value < 0.05. The enrichment analysis of the Kyoto Encyclopedia of Genes and Genomes (KEGG) pathway^11–13^ was accomplished using the hypergeometric test, and a q value < 0.05 indicated a significant pathway in the DEGs.

### Western blotting

The cells were lysed in RIPA buffer (Cat. No. AR0102; Boster, Wuhan, China), and a BCA protein assay kit was used to determine the protein concentration (Cat. No. P0012; Beyotime, Shanghai, China). The extracted protein was separated using SDS-PAGE and then transferred onto a PVDF membrane (Cat. No. IPVH00010; Millipore, USA). The membranes were blocked with TBST plus 5% skimmed milk for 1 h and subsequently incubated with primary antibodies (Table 1). After incubation with primary antibodies and washing with TBST, the membranes were incubated with anti-mouse IgG and anti-rabbit IgG. The protein bands were visualized using an ECL kit (Cat. No. G2014; Servicebio, Wuhan, China) and analyzed using ImageJ software.

**Table 1:** Antibodies used in Western blotting.

Antibody	Company	Cat. No	Dilution
PNO1	Proteintech	21059-1-AP	1:500
TGFB2	ABclonal	A3640	1:1000
BMP6	ABclonal	A4538	1:1000
YAP	ABclonal	A17075	1:1000
p-YAP	ABclonal	AP0489	1:1000
MST1	ABclonal	A8043	1:1000
p-MST1	ABclonal	AP0906	1:1000
LATS1	ABclonal	A17992	1:1000
p-LATS1	ABclonal	AP0904	1:1000
TAZ	ABclonal	A8202	1:1000
p-TAZ	ABclonal	AP0919	1:1000
SMAD2	ABclonal	A19114	1:1000
p-SMAD2	ABclonal	AP0269	1:1000
SMAD3	ABclonal	A19115	1:1000
p-SMAD3	ABclonal	AP0727	1:1000
GAPDH	Abclonal	A19056	1:5000
β-Actin	Santa Cruz	sc-47778/M	1:1000
HRP goat anti-mouse IgG	ZSGB-BIO	ZB-2305	1:5000
HRP goat anti-rabbit IgG	ZSGB-BIO	ZB-2301	1:5000

### Statistical analysis

GraphPad Prism 8.0.2 (GraphPad Software Inc., San Diego, CA, USA) was used for data analysis and visualization. *t* test was conducted to determine the difference between the two groups. The continuous data were presented as means ± standard deviation. All cell experiments were repeated at least three times, and a *P* value < 0.05 indicated a statistically significant difference (*P < 0.05, **P < 0.01, ***P < 0.001, ****P < 0.0001).

### Ethics approval and consent to participate

This study was carried out in accordance with the principles of the Basel Declaration. Our study was approved by The Medical Ethics Committee of the Third Hospital of Shandong province (approval no. KYLL-2022043). The samples were collected in accordance with the ethical standards of the Declaration of Helsinki. All patients provided written informed consent prior to enrollment in the study.

## Results

### Expression level of PNO1 in osteosarcoma samples

**Figure 1:**
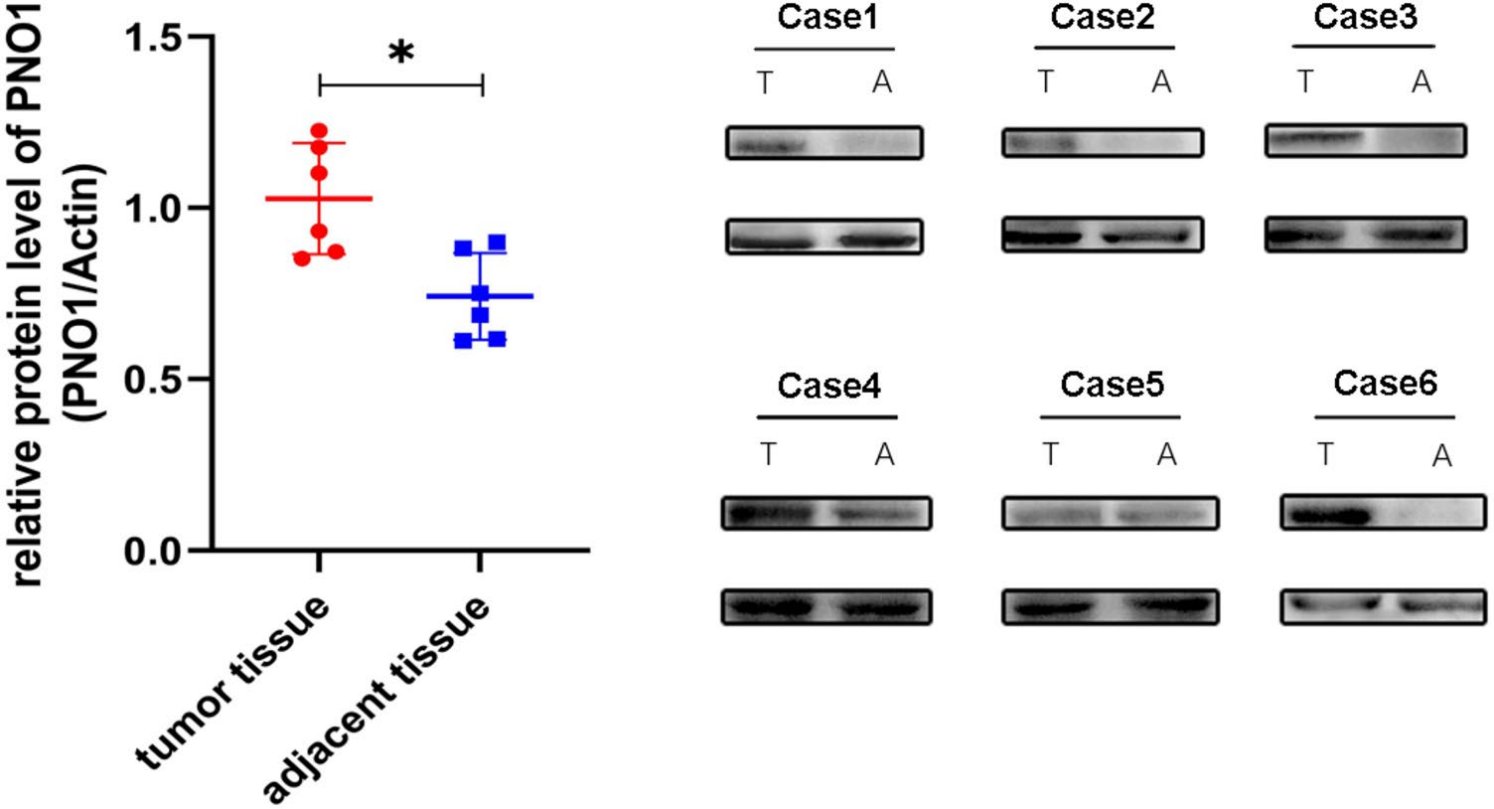
Protein expression of PNO1 in osteosarcoma samples (The bots were cut prior to hybridisation with antibodies, thus the images with adequate length were not provided).

Western blotting was adopted to investigate the expression of PNO1 in osteosarcoma. The result showed that, compared with adjacent tissue samples, the tumor tissues displayed a higher expression level of PNO1 (Fig. 1).

### Downregulating the expression of PNO1 inhibited the proliferation of osteosarcoma cells

**Figure 2:**
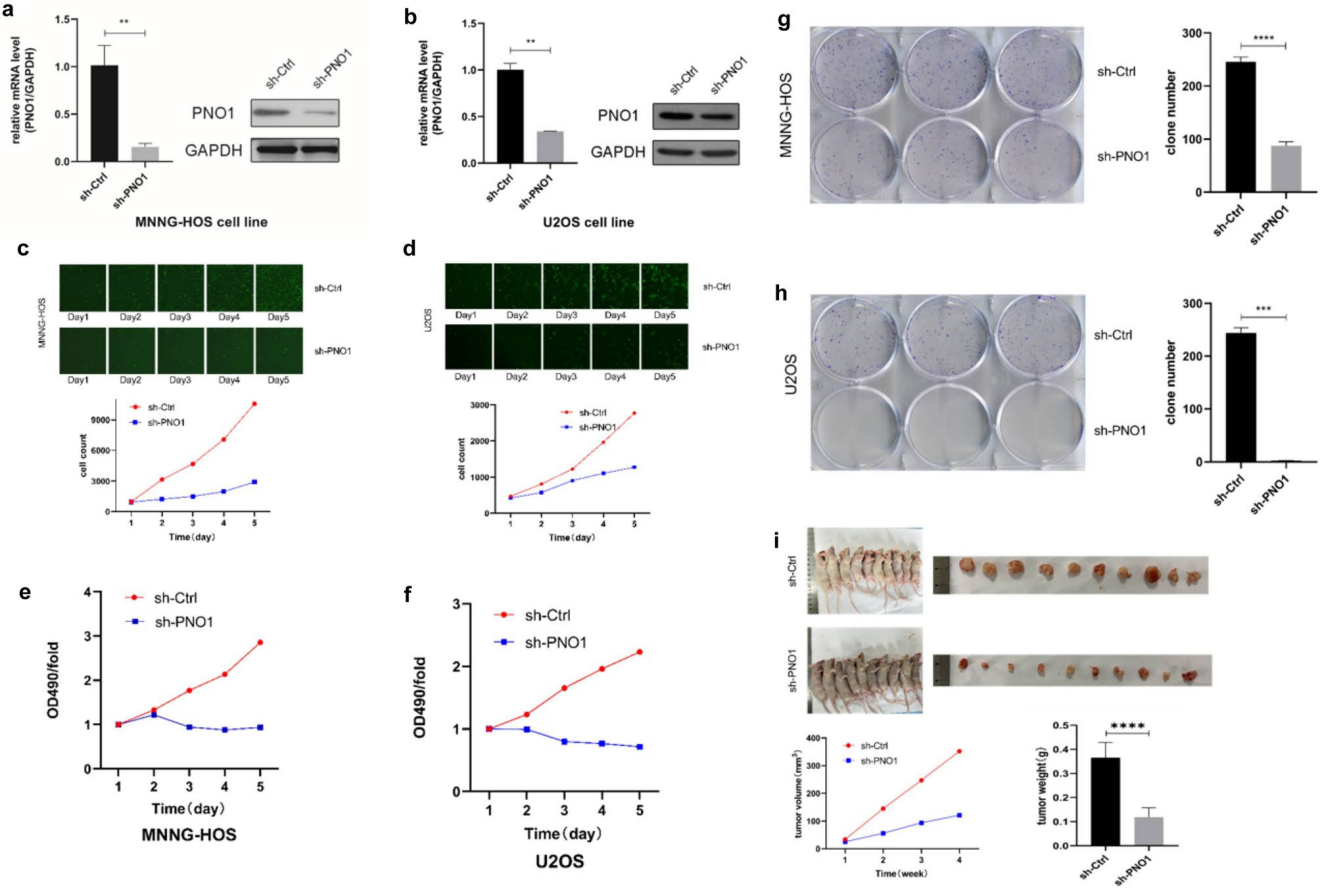
(**a**,**b**) The knockdown of PNO1 inhibited the proliferation of osteosarcoma cells. (**c**,**d**) Celigo cell counting assays of sh-PNO1 and sh-Ctrl cells. (**e**,**f**) MTT assays of sh-PNO1 and sh-Ctrl cells. (**g**,**h**) Colony formation assays of sh PNO1 and sh-Ctrl cells. (**i**) In vivo experiment of sh-PNO1 and sh-Ctrl cells.

We selected two osteosarcoma cell lines MNNG-HOS and U2OS to investigate the role of PNO1. Lentiviral plasmids were transfected for downregulating the expression of PNO1 (sh-PNO1) and empty vectors for control (sh-Ctrl) in two cell lines. The knockdown efficiency was confirmed by qRT-PCR and Western blotting. The results indicated that PNO1 mRNA levels were reduced by 84.8% and 66.2% in MNNG-HOS and U2OS cells, respectively (Fig. 2a and b).

Then, we examined the effect of knocking down PNO1 on cell proliferation in vitro using Celigo cell counting assay, MTT assay, and colony formation assay. Celigo cell counting assays indicated that the proliferation of sh-PNO1 cells was significantly suppressed (Fig. 2c and d). MTT assay showed that the viability of sh-PNO1 cells was inhibited compared with that of sh-Ctrl cells (Fig. 2e and f). The aforementioned effects were further confirmed using the colony formation assay (Fig. 2g and h).

For in vivo experiment, we selected MNNG-HOS to construct an osteosarcoma nude mouse model. Twenty BALB/c female nude mice were subcutaneously inoculated with MNNG-HOS cells, with 10 mice in each group (sh-PNO1 and sh-Ctrl). The in vivo experiment lasted for 4 weeks. The results of tumor size and weight are shown in Fig. 2i, suggesting that the mice in the sh-PNO1 group developed apparently smaller and lighter tumors compared with the mice in the sh-Ctrl group. These results indicated that knocking down PNO1 expression could inhibit the proliferation of osteosarcoma cells in vitro and in vivo.

### Low-level expression of PNO1 could reduce the migration and invasion of osteosarcoma cells

**Figure 3:**
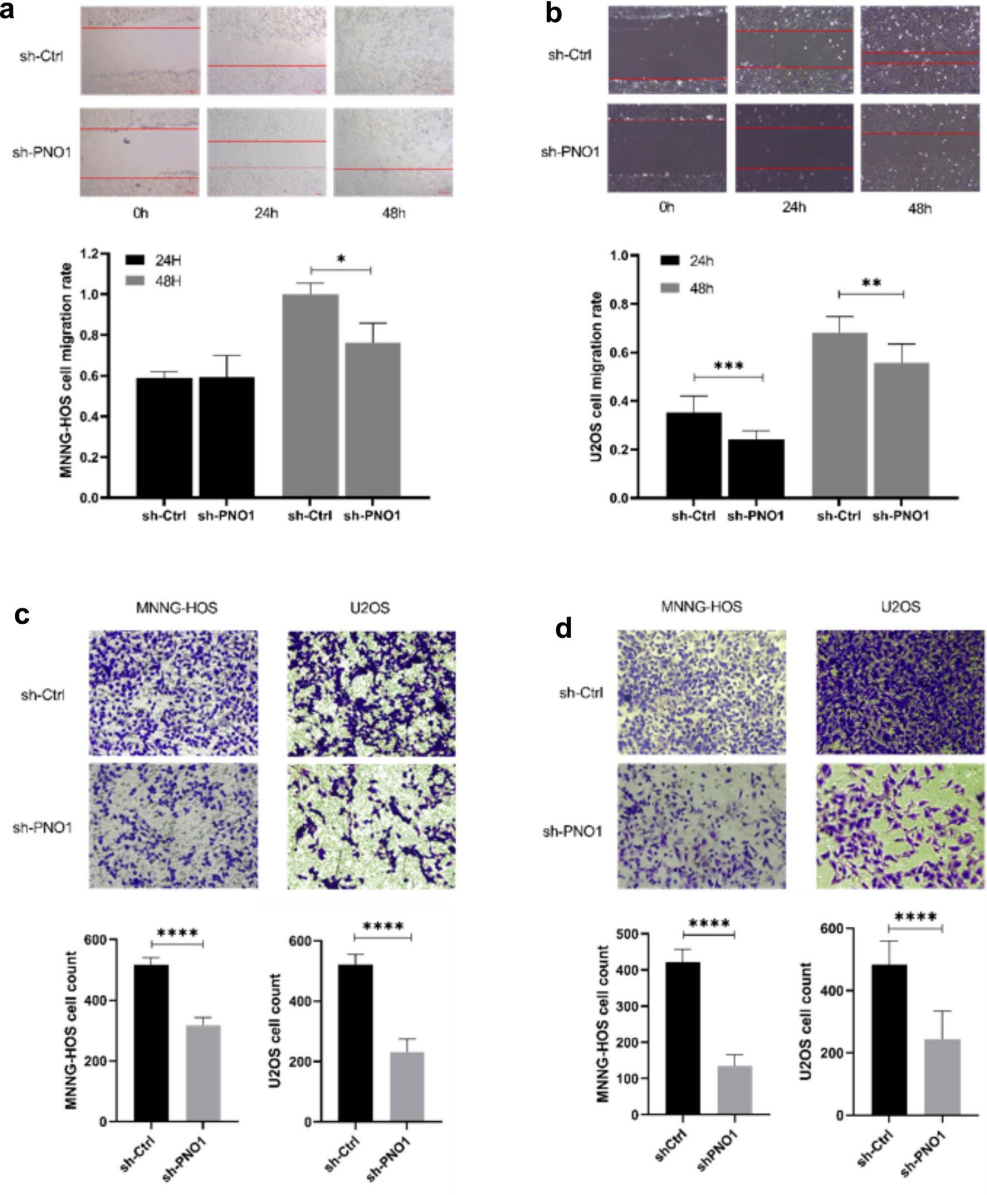
(**a**,**b**) Wound-healing assays of sh-PNO1 and sh-Ctrl cells. (**c**,**d**) Transwell assays of sh-PNO1 and sh-Ctrl cells.

Wound-healing and Transwell assays were used to investigate whether PNO1 knockdown would affect the migration and invasion abilities in osteosarcoma cells. The result of the wound-healing assay showed that the migration rate in the sh-PNO1 group was lower than that in the sh-Ctrl group (Fig. 3a and b). Moreover, Transwell assays also indicated that the number of migrating and invading cells was obviously lower in the sh-PNO1 group than in the sh-Ctrl groups (Fig. 3c and d).

### Knocking down the expression level of PNO1 could induce cell apoptosis and cell cycle arrest in U2OS cells

**Figure 4:**
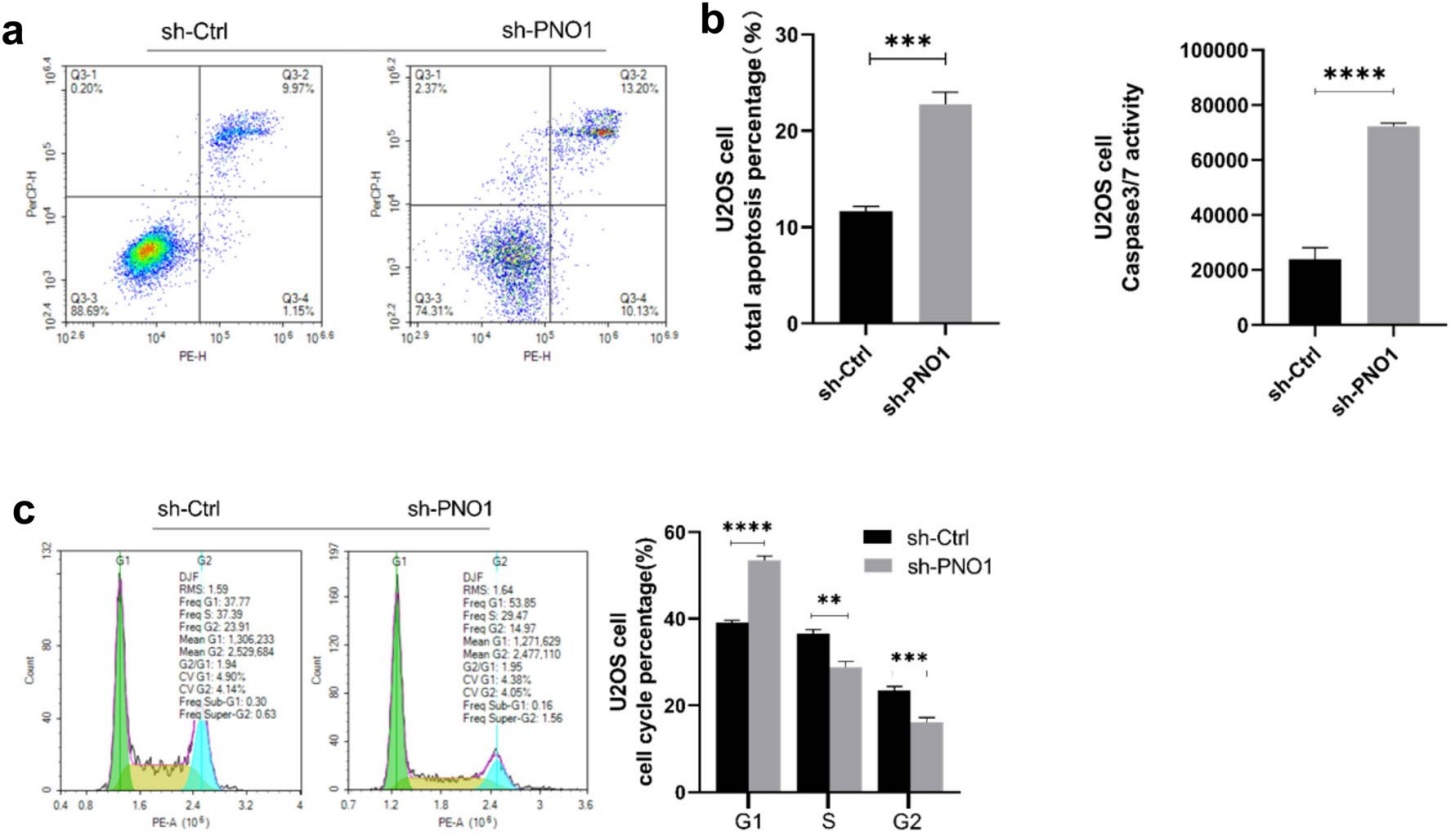
(**a**) Flow cytometry apoptosis analysis of sh-PNO1 and sh-Ctrl cells. (**b**) Caspase-3/7 activity assay of sh-PNO1 and sh-Ctrl cells. (**c**) Flow cytometry cell cycle analysis of sh-PNO1 and sh-Ctrl cells.

We performed flow cytometry and caspase-3/7 activity assay in U2OS cells in the sh-PNO1 and sh-Ctrl groups. The flow cytometry apoptosis analysis suggested that the apoptosis rate was 22.76% in the sh-PNO1 group and 11.65% in the sh-Ctrl group (Fig. 4a). Caspase-3/7 activity assay showed that caspase-3/7 activity obviously increased in the sh-PNO1 group (Fig. 4b). The results of the two assays revealed that inhibiting the expression of PNO1 promoted the apoptosis of U2OS cells. Besides, flow cytometry analysis indicated that PNO1 knockdown induced cell cycle arrest in the G0/G1 phase, which was associated with the decrease in the S and G2 phases of U2OS cells (Fig. 4c).

### PNO1 could regulate the progression of osteosarcoma via the TGF-β and YAP/TAZ signaling pathway

**Figure 5:**
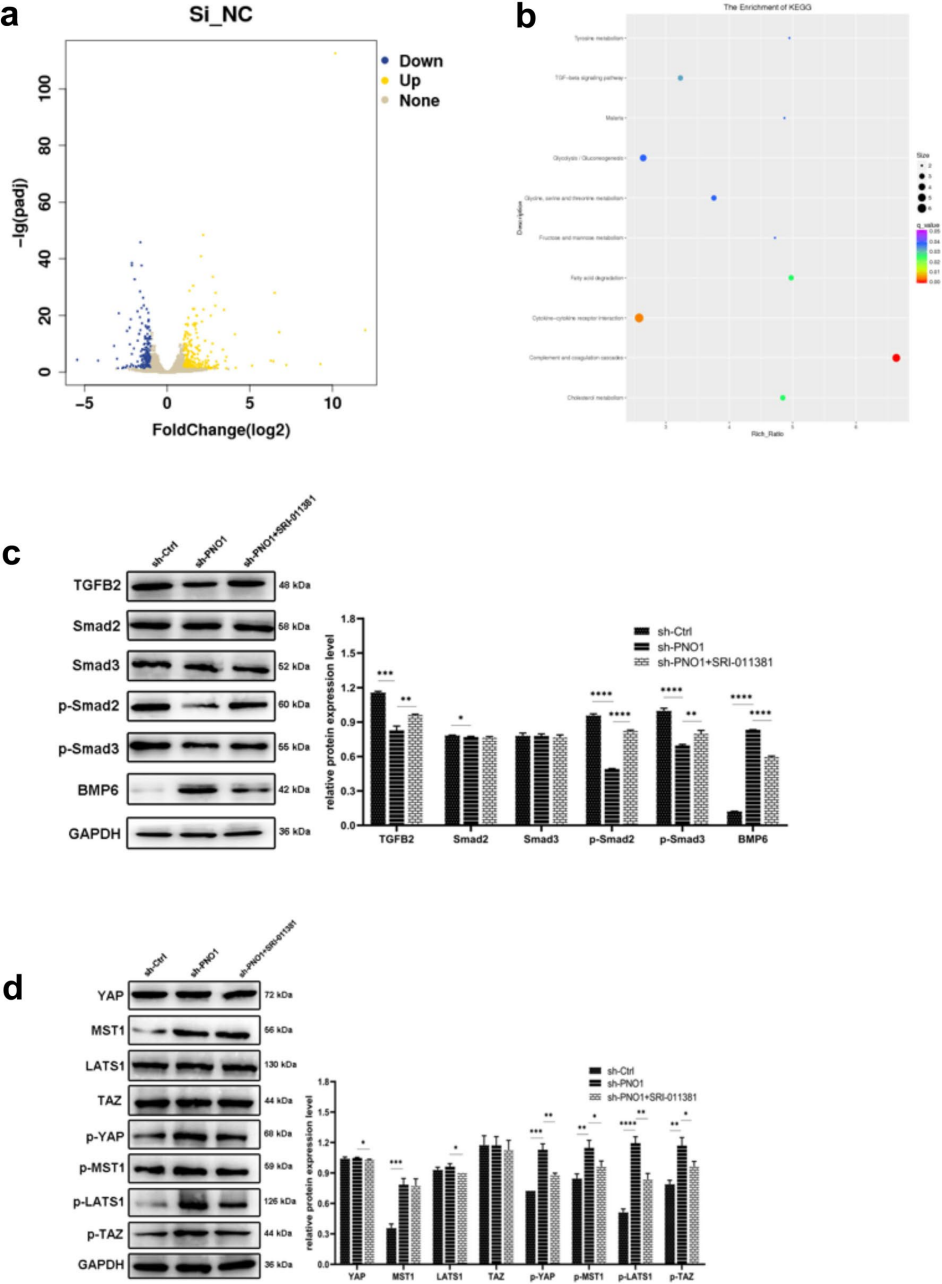
(**a**) DEGs identified between sh-PNO1 HOS and sh-Ctrl HOS cell groups. (**b**) KEGG pathway analysis of DEGs. (**c**,**d**) Western blotting of TGF-β and YAP-TAZ signaling pathways (The bots were cut prior to hybridisation with antibodies, thus the images with adequate length were not provided).

Transcriptome sequencing was performed in sh-PNO1 HOS and sh-Ctrl HOS cells to explore the signaling pathways associated mediated by PNO1. A total of 402 DEGs were obtained, including 234 upregulated genes and 168 downregulated genes (Fig. 5a).

KEGG pathway analysis revealed that DEGs were significantly correlated with complement and coagulation cascades, cytokine–cytokine receptor interaction, cholesterol metabolism, fatty acid degradation, and TGF-β signaling pathway (Fig. 5b).

Western blotting showed that the down-regulation of the PNO1 expression could inhibit the expression of TGFB2, Smad2, p-Smad2, and p-Smad3, but promote the expression of BMP6. Moreover, the expression of YAP/TAZ pathway related proteins were investigated, which exhibited a close correlation with TGF-β signaling. Interestingly, PNO1 expression loss increased the level of MST1 and LATS1, and promoted phosphorylation of MST1, LATS1, TAZ, and YAP (Fig. 5c and d). Moreover, the group with SRI-011381, a TGF-β signaling agonist, showed a reversed trend for the related proteins. Overall, these results indicated that PNO1 could regulate the progression of osteosarcoma cells via TGF-β and YAP/TAZ signaling pathway.

## Discussion

Recently, aberrant ribosome biogenesis, which requires various biogenesis factors, has been widely recognized as a specific hallmark in tumor cells^14^. Previous studies proved that PNO1, as a ribosome assembly factor, played an important role in cancer treatment and hence could be a potential target for cancer therapy^8,9,15^. However, the regulatory function and mechanisms of PNO1 in osteosarcoma were poorly known. In this research, we explained the oncogenic function and mechanisms of PNO1 in osteosarcoma. Analysis results demonstrated that the over-expression of PNO1 acted as contribution in malignant progression of osteosarcoma. Moreover, the oncogenic mechanisms of PNO1 was mediated by the pathways of YAP/TAZ and TGF-β.

PNO1 emerges as crucial function in carcinogenesis. In the current study, we found the found the increased expression of PNO1 in osteosarcoma tissues and cell lines. Moreover, the knockdown of PNO1 could inhibit proliferation and induce apoptosis and cell cycle arrest, displaying the inhibition of cell growth. All these results suggested the tumor promoting function of PNO1 in osteosarcoma. The expression manner of PNO1 was specific to the malignant potential of cancer cells, suggesting its predictive potential for human cancer. The study designed by Han et al. showed that the over-expression of PNO1 was a sign for dismal prognosis of hepatocellular carcinoma^16^. The expression of PNO1 was confirmed as a biomarker for survival of urinary bladder carcinoma^17^. In addition, PNO1 was designed as a prognostic biomarker for the patients with triple-negative breast cancer^18^ and glioma^19^. However, due tot he relatively small sample size and the lack of follow-up information, the predictive values of PNO1 for osteosarcoma were not investigated. Further analysis is required to address the issue.

Due to its unclear etiology, the clinical management of osteosarcoma still remains a great challenge^20^. Osteosarcoma is a complex malignancy which is driven by multiple genetic and non-genetic factors^21^. With the development of sequencing techniques, to investigate the regulatory function and mechanisms mediated by genetic factors becomes an effective approach to provide predictive biomarkers and therapeutic targets for cancer patients. In order to expound the oncogenic mechanisms of PNO1 in osteosarcoma, transcriptome sequencing and bioinformatic analysis was used in our study. We found that the downregulated expression of PNO1 resulted in the suppression of TGFB2, Smad2, p-Smad2, and p-Smad3, and upregulated the expression of BMP6. Meanwhile, the levels of TGFB2, p-Smad2, p-Smad3, and BMP-6 showed a reverse trend after the SRI-011381 application, suggesting that PNO1 could influence the progression of osteosarcoma cells via the TGF-β signaling pathway. TGF-β signaling pathway plays crucial roles in tumorigenesis through mediating the malignancy phenotype of cancer cells, epithelial-mesenchymal transitions (EMTs), immune escape, etc.^22,23^. Growing evidences suggested the promoting function of TGF-β/Smad signaling pathway in osteosarcoma^24,25^. Meanwhile, we found the opposite regulation of PNO1 on TGF-β and BMP pathways. TGF-β and BMP pathways were reported to induce antagonistic effects in several malignant tumors^26^. This conclusion might explain the results in our study. PNO1 could contribute to malignant progression of osteosarcoma by activating TGF-β pathway and suppressing BMP pathways.

YAP/TAZ pathway is key downstream component of Hippo signaling pathway, which can be activated and phosphorylated by upstream MST1/2 and LATS1/2, leading to the inhibition of function, causing cell proliferation and tumor growth^27^. The location of YAP/TAZ proved that they could be associated with the activation of Smad2/3 complex. Inhibited phosphorylation of YAP/TAZ led to the accumulation of YAP/TAZ and Smad2/3 in the nucleus and promoted EMT of cancer cells^28^. Several studies showed that YAP was closely correlated with the proliferation of osteosarcoma cells, while TAZ was more likely to be involved in metastasis, invasion, and EMT^29^. In this study, we found that the deletion of PNO1 promoted the phosphorylation of MST1, LATS1, YAP, and TAZ, but the change in YAP and TAZ levels was not significant. Moreover, the phosphorylation of YAP/TAZ signaling protein was suppressed after SRI-011381 treatment. These changes indicated that PNO1 might play a role in the phosphorylation of YAP/TAZ signaling pathway in osteosarcoma, but it did not affect the level of YAP and TAZ. Further, the activation of TGF-β signaling pathway could reverse the phosphorylation of YAP/TAZ signaling induced by the down-regulation of PNO1. PNO1 might change the interaction of TGF-β pathway with YAP/TAZ complex.

This study had several limitations. First, small sample size were used in this study due to the low incidence of osteosarcoma. A multicenter research would be an effective approach to recruit more patients to strenghten our findings and explore potential clinical significance of it. In addition, the data from the online databases were be valuable to verify the clinical performance of PNO1 in osteosarcoma. Second, the transcriptome sequencing and bioinformatics analysis was performed for only one cell line. In view the heterogeneity across different types of osteosarcoma cell lines, the results might have bias. Further analyses based on multiple cell lines or online databases are necessary to verify our results. Third, we verified that PNO1 influenced osteosarcoma via TGF-β and YAP/TAZ signaling pathways, but the level of change in the related proteins was not consistent. This abnormalities might be attributed to the complex interactions between the signaling pathways. Both of TGF-β and YAP/TAZ pathways are complex, which are regulated by various molecules in tumorigenesis. PNO1 might only damage one interacting mechanism, and TGF-β and YAP/TAZ might interact through other compensation mechanisms. Therefore, further molecular studies are still necessary to explore the complex regulatory function of PNO1 in osteosarcoma.

Taken together, this study verifies the tumor promoting effect and mechanisms of PNO1 in osteosarcoma. PNO1 may alter the progression of osteosarcoma via TGF-β and YAP/TAZ signaling pathways. PNO1 could serve as a therapeutic target of osteosarcoma (Supplementary Figs. 1–4).

### Supplementary Information

Supplementary Figures.

## Data Availability

All data generated or analyzed during this study are included in this published article.

## Abbreviation

PNO1: Partner of NOB1
