# Supplementary material for: PNO1 promotes the progression of osteosarcoma via TGF-β and YAP/TAZ pathway
Source: Sci Rep. 2023 Dec 9;13:21827. doi: 10.1038/s41598-023-49295-8 (PMC10710495; doi:10.1038/s41598-023-49295-8)
Supplement: Supplementary file 1 — Supplementary Figures. [file 41598_2023_49295_MOESM1_ESM.pdf]

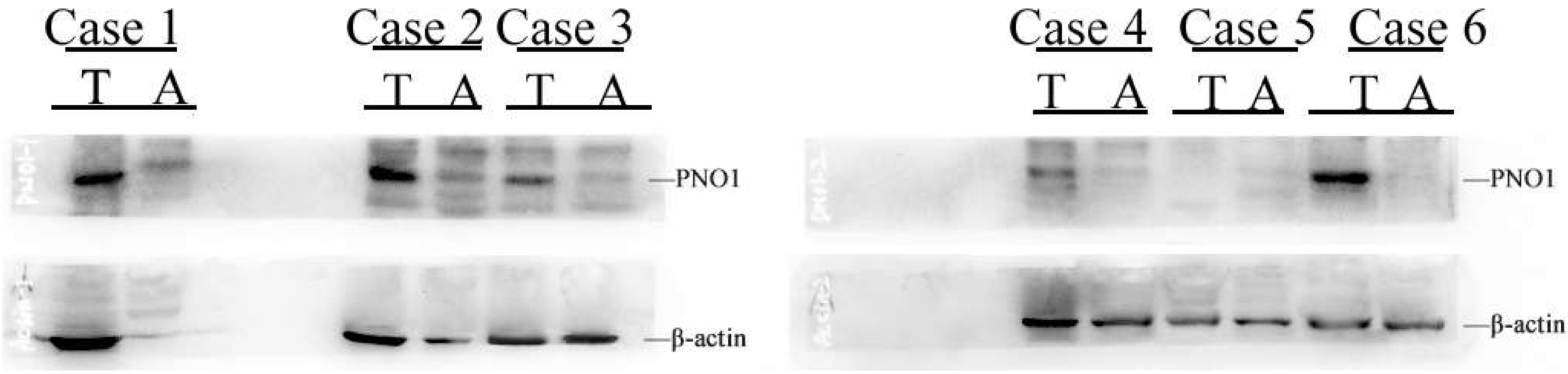

Supplementary figure 1. The original western blot images to show PNO1 in six pairs of osteosarcoma samples. T: tumor; A: adjacent normal tissues.

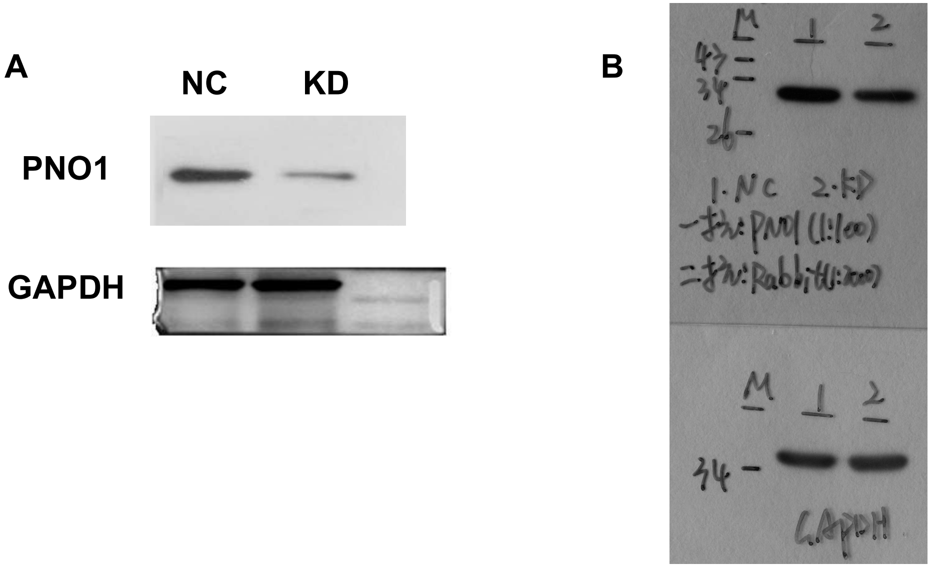

Supplementary figure 2. The original images to show PNO1 expression in MNNG-HOS (**A**) and U2OS (**B**) cells transfected by sh-Ctrl and sh-PNO1, respectively. NC: sh-Ctrl; KD: sh-PON1

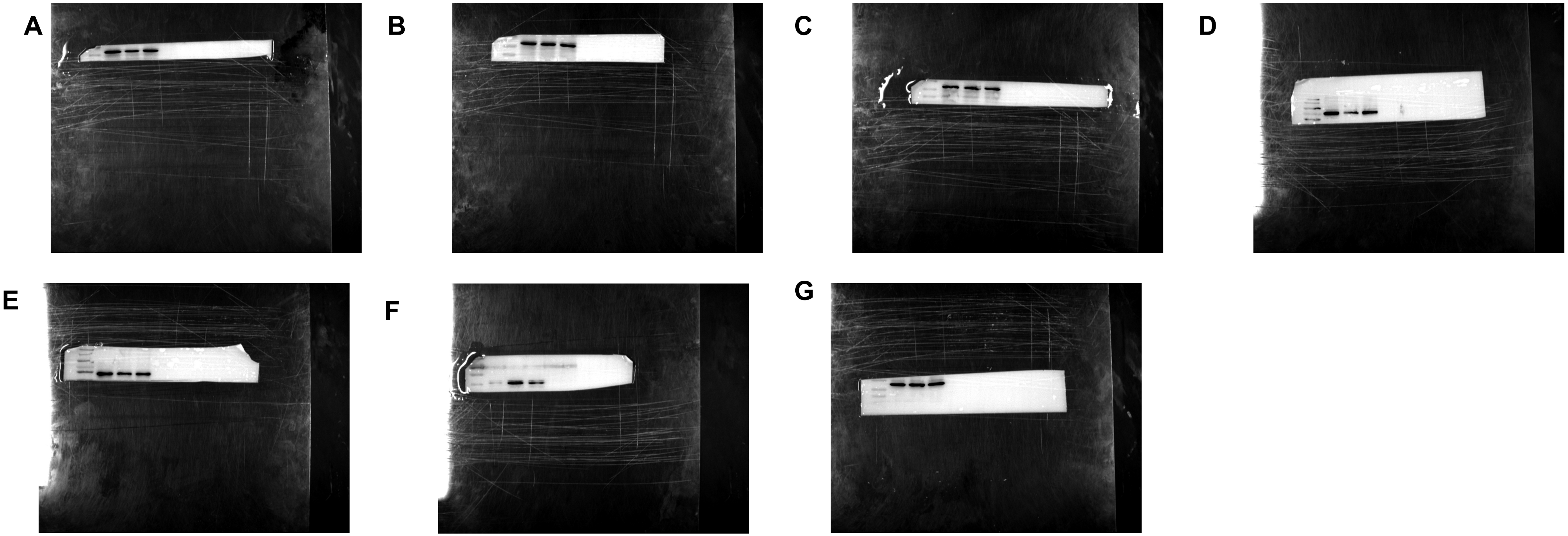

Supplementary figure 3. The original images to show the expression of TGF-β pathway related proteins (A: TGFB2; B: Smad2; C: Smad3; D: p-Smad2; E: p-Smad3; F: BMP6; G: GAPDH) after the knockdown of PNO1 or the treatment of SRI-011381. Lane 1: Marker; Lane 2: si-Ctrl; Lane 3: sh-PNO1; Lane 4: sh-PNO1+SRI-011381; SRI-011381 is a TGF-β signaling agonist.

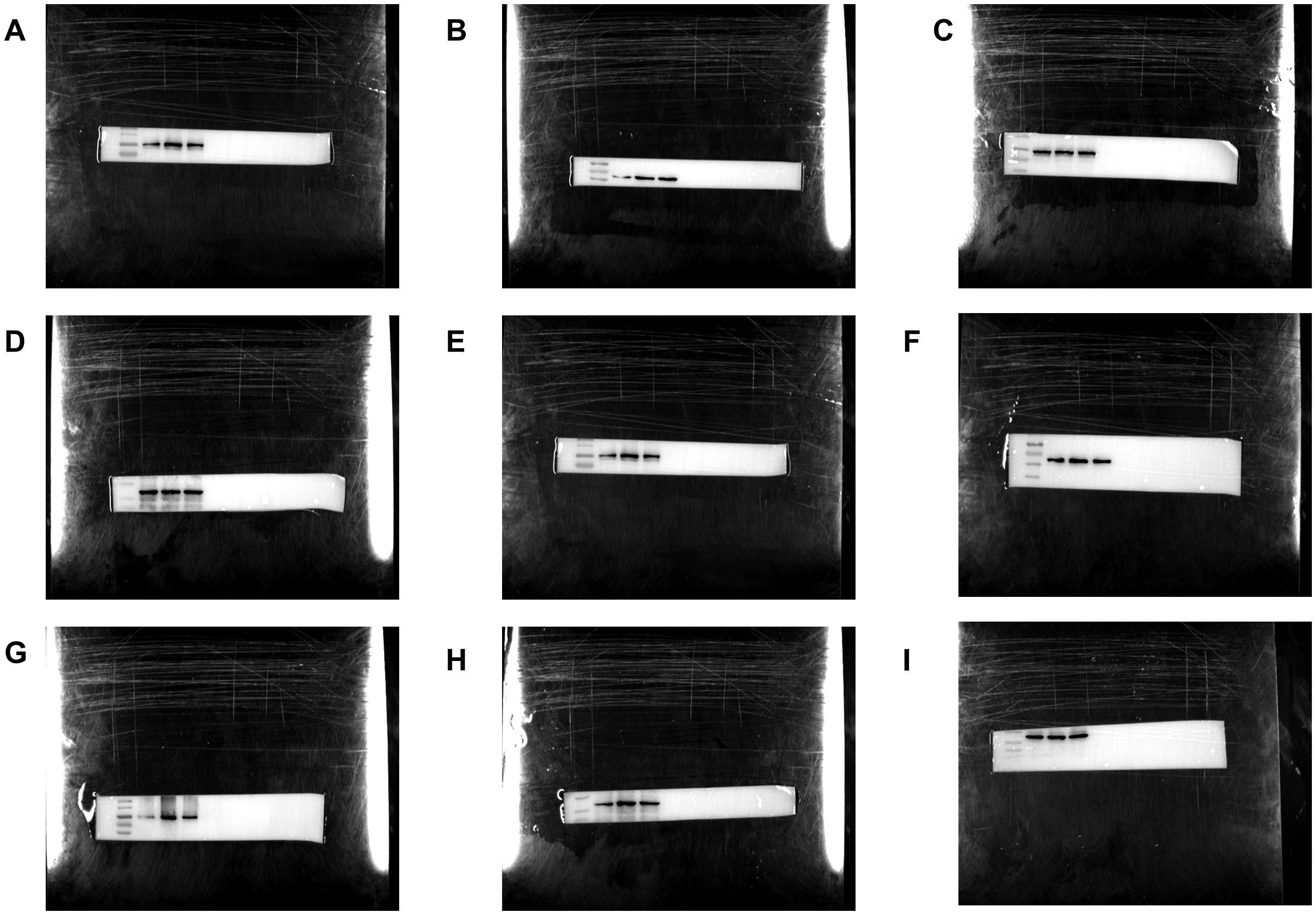

Supplementary figure 4. The original images to show the expression of YAP/TAZ pathway related proteins (A: YAP; B: MST1; C: LATS1; D:TAZ; E: p-YAP; F: p-MST1; G: p-LAST1; H: p-TAZ; I: GAPDH)after the knockdown of PNO1 or the treatment of SRI-011381. Lane 1: Marker; Lane 2: si-Ctrl; Lane 3: sh-PNO1; Lane 4: sh-PNO1+SRI-011381; SRI-011381 is a TGF-β signaling agonist.
